# LMO2 regulates STAT3 localization to the nucleus in breast cancer cells

**DOI:** 10.17912/micropub.biology.001928

**Published:** 2026-07-30

**Authors:** Isobel J Fetter, Veronica Haro Acosta, Sachin Parecadan, Shaheen S Sikandar

**Affiliations:** 1 Molecular, Cell & Developmental Biology, UC Santa Cruz, Santa Cruz, CA, United States; 2 Institute for the Biology of Stem Cells, UC Santa Cruz, Santa Cruz, CA, United States; 3 Genomics Institute, UC Santa Cruz, Santa Cruz, CA, United States

## Abstract

STAT3 phosphorylation and transcriptional activity promote breast cancer growth and metastasis. We have previously reported that the transcriptional adaptor LMO2 is required for metastasis in breast cancer and promotes STAT3-JAK2 interaction, leading to STAT3 phosphorylation in metastasis-initiating cells. Here, we find that constitutively activated STAT3 is insufficient to drive metastasis in the absence of LMO2. Mechanistically, we find that LMO2 is required not only for STAT3 phosphorylation in the cytoplasm but also for STAT3 translocation to the nucleus. These data suggest that LMO2 promotes STAT3 activation and localization to the nucleus in breast cancer cells.

**Figure 1. LMO2 regulates STAT3 localization to the nucleus in breast cancer cells:** **(A)** Schematic of constitutively active STAT3 (aSTAT3) and sh *LMO2* expression in MDA-MB-468 cells followed by orthotopic transplant in NSG mice to evaluate tumor burden and metastasis. **(B)** Immunoblotting of MDA-MB-468 cells transduced with either control (pSicoR) or aSTAT3 plasmid. **(C)** Tumor weight in control (pRSI12/pSicoR), sh *LMO2* (sh *LMO2* /pSicoR), aSTAT3 (pRSI12/aSTAT3), and sh *LMO2* /aSTAT3 tumors generated from MDA-MB-468 cell xenografts. **(D)** Representative fluorescent images of spontaneous RFP+/GFP+ lung metastases from mice with control, sh *LMO2* , aSTAT3, and sh *LMO2* /aSTAT3 tumors in (B). **(E)** Quantification of metastases in (D). For C-E, n = 5 mice per group. **(F)** Representative immunoblot of cytoplasmic and nuclear fractions of MDA-MB-468 cells transduced with control (pRSI12/pSicoR), sh *LMO2* (sh *LMO2* /pSicoR), aSTAT3 (pRSI12/aSTAT3), and sh *LMO2* /aSTAT3 plasmids, probed for phospho-STAT3 (Tyr705), STAT3, FLAG, Lamin B, and β-Tubulin 1. Densitometric values for each representative blot are below the respective band. **(G)** Quantification of cytoplasmic phospho-STAT3 (Tyr705) as shown in (F), normalized to β-Tubulin 1 cytoplasmic loading control. **(H)** Quantification of nuclear phospho-STAT3 (Tyr705) as shown in (F), normalized to Lamin B nuclear loading control. For G-H, n = 5 biological replicates. **(I)** Quantification of cytoplasmic FLAG as shown in (F), normalized to β-Tubulin 1 cytoplasmic loading control. **(J)** Quantification of nuclear FLAG as shown in (F), normalized to Lamin B nuclear loading control. For I-J, n = 3 biological replicates. Data shown as mean ± standard deviation. Statistical significance was calculated with ordinary 1-way ANOVA with multiple comparisons (C, E, G-H) and unpaired t-tests (I-J).

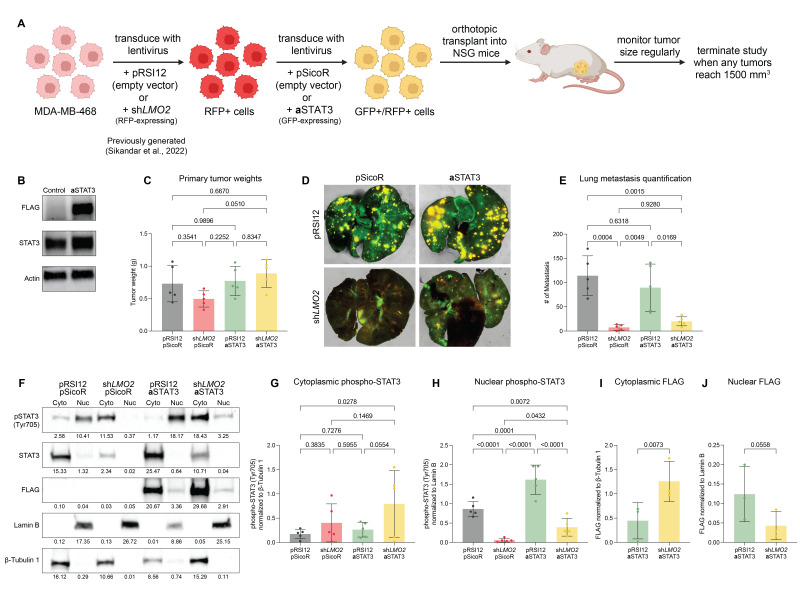



## Description

Metastasis is the leading cause of breast cancer-related deaths, making it imperative to develop strategies to target metastasis (Dillekås et al., 2019; Siegel et al., 2025). It is well established that breast tumors contain heterogeneous populations, with only a small subset of tumor cells exhibiting tumor-initiating and metastatic capacity (Lawson et al., 2015; Celià-Terrassa and Kang, 2016; Sikandar et al., 2017). Understanding molecular mechanisms regulating metastasis-initiating cells (MICs) will broaden potential targets and treatment strategies for breast cancer.

Previous studies have demonstrated that inflammatory cytokines such as IL6 and TNFα activate the JAK/STAT and NFκB pathways, resulting in transcriptional changes leading to tumorigenesis and metastasis (Coussens and Werb, 2002; Quail and Joyce, 2013; Hibino et al., 2021). STAT3 is phosphorylated by JAK2, homodimerizes to move to the nucleus and regulate transcription of its target genes, promoting proliferation, survival, and self-renewal (Schindler and Darnell, 1995).

We recently identified a population of MICs in breast cancer that express LMO2, a transcriptional adaptor protein in hematopoietic stem cells and a T-cell oncogene (Yamada et al., 1998; McCormack et al., 2010). *LMO2* knockdown did not affect the growth of primary tumors, but significantly reduced the number of circulating tumor cells and lung metastases (Sikandar et al., 2022). Mechanistically, we found that LMO2 binds to the transcription factor STAT3 and is required for STAT3 phosphorylation in breast cancer cells (Sikandar et al., 2022). Consistent with our previous findings, another study showed LMO2 activates STAT3 signaling in glioblastoma to increase stem-like activity of cancer cells, suggesting that LMO2 activates STAT3 signaling in multiple cancers (Park et al., 2022). Here, we further investigate interactions between LMO2 and STAT3 during breast cancer metastasis by combining constitutively activated STAT3 with the loss of LMO2.

STAT3 is constitutively activated in several cancers, including breast cancer (Kim et al., 2012; Carpenter and Lo, 2014; Galoczova et al., 2018; J.-H. Ma et al., 2020). To test whether constitutively active STAT3 (aSTAT3) is sufficient to rescue the decreased metastatic potential observed upon *LMO2* knockdown, we first obtained a FLAG-tagged aSTAT3 construct (Hillion et al., 2008) and confirmed its expression by immunoblotting ( **Fig. 1B** ). We then generated four cell lines combining LMO2 knockdown and aSTAT3 and their respective controls (Ventura et al., 2004). These cell lines were orthotopically transplanted into the mammary fat pad of female NSG mice, and tumor growth was monitored over time ( **Fig. 1A** ). We found that there is no difference in tumor growth among the control, sh *LMO2* , aSTAT3, and aSTAT3/sh *LMO2* ( **Fig. 1C** ). This is consistent with our previous findings that *LMO2* knockdown significantly reduced metastasis, but did not impact tumor growth (Sikandar et al., 2022). We also found that aSTAT3 did not increase the number of metastases ( **Fig. 1D- E** ). Importantly, mice with shLMO2/aSTAT3 tumors have reduced metastasis similar to the mice with *LMO2* knockdown tumors, suggesting that the combination of constitutively active STAT3 and *LMO2* knockdown is insufficient to rescue metastasis ( **Fig. 1D- E** ).

As LMO2 is an adaptor protein and facilitates the formation of transcription factor complexes (Yamada et al., 1998), we next wanted to understand whether LMO2 is required for STAT3 localization to the nucleus. Hence, we performed subcellular fractionation and immunoblotting to find that, as expected, pSTAT3 is primarily located in the nucleus in the control and aSTAT3 cell lines ( **Fig. 1F** ). We observed an increase in cytoplasmic pSTAT3 in the sh *LMO2* /aSTAT3 cells compared to the control, suggesting that while the aSTAT3 construct increased pSTAT3, a significant amount of pSTAT3 remains in the cytoplasm and does not translocate to the nucleus ( **Fig. 1G** ). Indeed, in the nuclear fractions of both the sh *LMO2* and sh *LMO2* /aSTAT3 conditions, there is a significant reduction in nuclear pSTAT3 compared to the control conditions ( **Fig. 1H** ). Because the aSTAT3 construct can dimerize in absence of phosphorylation and translocate to the nucleus (Hillion et al., 2008), we also probed for FLAG-tag to determine whether LMO2 modulates translocation of aSTAT3. While FLAG-tagged STAT3 is detected in both the cytoplasm and nucleus of aSTAT3 and sh *LMO2* /aSTAT3 cells ( **Fig. 1F )** , the amount of aSTAT3 (as measured by FLAG expression) is significantly higher in the cytoplasm and reduced in the nucleus in sh *LMO2* /aSTAT3 cells as compared to aSTAT3 control ( **Fig. 1I, J** ). This suggests that LMO2 regulates STAT3 nuclear localization, even when STAT3 is constitutively active. In summary, these data suggest a novel role for LMO2 in breast cancer cells; in addition to stabilizing JAK2-STAT3 interactions to promote phosphorylation (Sikandar et al., 2022), LMO2 actively facilitates STAT3 localization to the nucleus.

STAT3 phosphorylation and nuclear localization are associated with tumorigenesis and metastasis in multiple cancers, with increased nuclear STAT3 leading to reduced overall survival in patients (Huang et al., 2017; Chen et al., 2023; Zhang et al., 2024). STAT3 can localize to the nucleus without phosphorylation, but phosphorylation is required for STAT3’s transcription factor activity (Liu et al., 2005). We observed a significant reduction in nuclear pSTAT3 in sh *LMO2* and sh *LMO2* /aSTAT3 conditions ( **Fig. 1H** ), suggesting that LMO2 promotes STAT3’s nuclear localization and thus its transcriptional activity. STAT3 is imported into the nucleus primarily through importin-α3 (KPNA4) or importin-α5 (KPNA1) in association with importin-β1 (KPNB1) (Liu et al., 2005; J. Ma and Cao, 2006). Some FLAG-tagged STAT3 is still found in the nucleus when *LMO2* is knocked down but significantly reduced compared to control cells ( **Fig. 1J** ). Structurally, LMO2 is composed of 2 LIM fingers, each containing 2 zinc finger motifs (El Omari et al., 2011). Interestingly, other zinc finger-containing proteins such as EZI (Nakayama et al., 2002), ZBP-89 (Wu et al., 2004), KLF4 (Qin et al., 2013), and IKZF1 and IKZF3 (Read et al., 2017) have been shown to associate with and modulate nuclear localization of STAT3.

STAT3 signaling is an appealing target for treating cancer. Multiple STAT3 inhibitors have been investigated in clinical trials with various efficacies and tolerances (Hong et al., 2013; Ogura et al., 2015; Oh et al., 2015; Jonker et al., 2016; Tolcher et al., 2018; Feldman, 2024), but there are currently no FDA-approved therapies targeting STAT3. Inhibiting STAT3 signaling can lead to off-target toxicity, so targeting STAT3 only in cancer cells could improve outcomes. Since the oncogenic effects depend on STAT3’s nuclear localization, disrupting its nuclear import represents an alternative therapeutic strategy. Disruption of LMO2-STAT3 interactions using newly developed inhibitors for LMO2 could prevent nuclear accumulation and suppress STAT3-driven transcription (Milton-Harris et al., 2020; Bery et al., 2021). Therapeutically, this approach offers a potential strategy to selectively target constitutively active STAT3 in tumors while sparing transient STAT3 activation in normal tissues. However, it is also possible that LMO2 has additional functions independent of its interaction with STAT3 that contribute to metastasis. Future structure-function studies will determine how LMO2 promotes STAT3 localization to the nucleus in metastasis-initiating cells and whether LMO2 binds to additional molecules to modulate metastasis in breast cancer.

## Methods

**Cell Line Generation**

MDA-MB-468 and HEK293T cells were obtained from the American Type Culture Collection. Cells were cultured in DMEM supplemented with penicillin (120 μg/mL), streptomycin (100 μg/mL), amphotericin B (0.25 μg/mL), and 10% FBS. LMO2 shRNA targeting the 3′UTR (5′-GACGCATTTCGGTTGAGAA-3′) was cloned into the pRSI12-U6-(sh)-HTS4-UbiC-TagRFP-2A-Puro shRNA expression vector (Cellecta). The pRSI12 empty vector was used as a control. EF.STAT3C.Ubc.GFP was a gift from Linzhao Cheng (Addgene plasmid #24983; http://n2t.net/addgene:24983; RRID: Addgene_24983). pSicoR was a gift from Tyler Jacks (Addgene plasmid #11579; http://n2t.net/addgene:11579; RRID: Addgene_11579) and was used as an empty vector control for the aSTAT3 plasmid. Viruses were produced in HEK293T cells using the second-generation lentiviral system and transfection using Lipofectamine 2000 (Life Technologies). Supernatants were collected at 48 and 72 hours, filtered with a 0.45-μm filter, and precipitated with lentivirus precipitation solution (Alstem LLC) per the manufacturer’s instructions. Viral titers were determined by flow cytometry analyses of HEK293T cells infected with serial dilutions of concentrated virus.

**Mouse Models**

*NOD.Cg-Prkdc ^scid^ Il2rg ^tm1Wjl^ /SzJ* (NSG) (stock #005557) mice were purchased from The Jackson Laboratory and bred at the University of California Santa Cruz (UCSC). All mice used for this study were maintained at the UCSC Animal Facility in accordance with the guidelines set forth by UCSC and the Institutional Animal Care and Use Committee (Protocol number SIKAS2010).

**Xenografts**

To prepare cells for orthotopic injection, cells were trypsinized with standard protocols, and live cell counts were assessed by Trypan Blue staining. Each cell line was resuspended in DMEM containing 40% Matrigel and subcutaneously injected into the fourth abdominal fat pads on one side of female NSG mice. 100,000 cells were used for each injection. Mice were monitored every week for tumor growth. All mice were euthanized if tumor growth reached the endpoint (1500 mm ^3^ ). Tumor size was measured using digital calipers. Tumors were harvested and weighed, and lungs were harvested and placed in PBS on ice. The lungs were then imaged in brightfield, RFP, and GFP on a Keyence Biorevo BZ-9000 digital widefield microscope. The RFP and GFP channels were then merged with FIJI (Schindelin et al., 2012), and RFP/GFP double positive lung metastases were manually counted.

**Subcellular fractionation**

Whole cell lysates were generated by lysis with radioimmunoprecipitation assay (RIPA) buffer, along with protease and phosphatase inhibitor cocktails (Thermo Fisher Scientific). Subcellular fractionation was performed as described previously (Baghirova et al., 2015; Rubio et al., 2024). Briefly, cultured cells were washed with room temperature PBS before detachment with trypsin. Cells were resuspended in DMEM with 10% FBS and pelleted at 500 x g. Increasingly strong detergents were used to lyse the cells, and the cytoplasmic fraction, the membrane-bound organelle fraction, and finally the nuclear fraction were collected from the supernatant. After the supernatant was collected for each fraction, the remaining cell material was washed 3 times with the respective lysis buffer to remove any remaining protein before the next lysis step. Protein concentration was measured with the Pierce BCA Protein Assay Kit (Thermo Scientific).

**Western Blotting**

For each sample, 20 μg of protein lysate was added to 10 μL 4x NuPAGE LDS buffer with 4% β-mercaptoethanol, then boiled at 95°C for 5 minutes before gel electrophoresis. SDS-PAGE gels were run at 100 V for 70 min, then transferred onto polyvinylidene difluoride membranes (Millipore) at 70 V for 90 min. Membranes were blocked with 5% BSA/Tris-buffered saline–Tween 0.1% (TBST) for 1 hour at RT and then subsequently probed with primary antibodies diluted at 1:1000 in 5% BSA/Tris-buffered saline–Tween 0.1% (TBST) overnight at 4°C. Membranes were then incubated in secondary antibodies containing HRP for 1 hour at RT. Clarity Western ECL Substrate (Bio-Rad) was used to enable visualization on the Bio-Rad ChemiDoc. Washes in between incubations were done for 10 min × 3 using TBST.

**Immunoblot Quantification**

Western blot bands were quantified using FIJI (Schindelin et al., 2012) software and normalized to controls as indicated in the figure legend. Rectangular regions of interest were drawn over lanes, then the peaks were plotted using the “Plot Lanes” tool. Vertical lines were then drawn to define peaks of interest, then the wand tool was used to measure the size of each peak. Densitometric values for phospho-STAT3 and FLAG were normalized to values for β-Tubulin 1, or Lamin B as noted in figure legends. Each sample was normalized to the loading control in the same lane. For example, the cytoplasmic phospho-STAT3 value for the control condition was divided by the β-Tubulin 1 value for the control on the same gel.

**Statistical Analysis**

All graphs show the mean, and error bars indicate standard deviations. P values are calculated using ordinary 1-way ANOVA with multiple comparisons and unpaired t-tests with GraphPad Prism. For animal studies, sample size was not predetermined to ensure adequate power to detect a prespecified effect size, no animals were excluded from analyses, and investigators were not blinded to group allocation during experiments.

## Reagents

**Table:** 

**Cell Line**	**Genotype**	**Available From**
MDA-MB-468	*Homo sapiens* , isolated from a pleural effusion of a female patient with metastatic adenocarcinoma of the breast	ATCC
HEK293T	*Homo sapiens,* derived from embryonic kidney 293 cells, contains SV40 T-antigen	ATCC

**Table:** 

**Plasmid**	**Description**
pSicoR	pSicoR was a gift from Tyler Jacks (Addgene plasmid #11579; http://n2t.net/addgene:11579; RRID: Addgene_11579)
aSTAT3	EF.STAT3C.Ubc.GFP was a gift from Linzhao Cheng (Addgene plasmid #24983; http://n2t.net/addgene:24983; RRID: Addgene_24983)
pRSI12	pRSI12-U6-(sh)-HTS4-UbiC-TagRFP-2A-Puro shRNA expression vector (Cellecta)
sh *LMO2*	LMO2 shRNA targeting the 3′UTR (5′-GACGCATTTCGGTTGAGAA-3′) was cloned into the pRSI12 shRNA expression vector (Cellecta)

**Table:** 

**Animal Strain**	**Genotype**	**Available From**
NSG *Mus musculus*	NOD.Cg- *Prkdc ^scid^ Il2rg ^tm1Wjl^ /* Szj	Jackson Laboratory (stock #005557)

**Table:** 

**Antibody**	**Animal and Clonality**	**Immunogen**	**Concentration**
anti-FLAG	Rabbit polyclonal (Sigma-Aldrich #F7425)	DYKDDDDK	1:2500
anti-STAT3	Mouse monoclonal, clone 9D8 (Abcam #ab119352)	peptide within human STAT3 aa 650 to the C-terminus	1:1000
anti-pSTAT3 (Y705)	Rabbit monoclonal, clone D3A7 (Cell Signaling Technology #9145)	synthetic phosphopeptide corresponding to residues surrounding Tyr705 of mouse Stat3	1:1000
anti-Lamin B	Goat polyclonal (Santa Cruz Biotechnology #sc-6216)	peptide mapping at the C-terminus of human Lamin B1	1:1000
anti-β-Tubulin 1	Mouse monoclonal, clone SAP.4G5 (Sigma-Aldrich #T7816)	synthetic peptide corresponding to the C-terminal sequence of β-tubulin isotype I	1:1000
anti-β-Actin, HRP conjugated	Mouse monoclonal, clone BA3R (Invitrogen #MA5-15739-HRP)	β-actin N-terminal peptide	1:5000
anti-mouse IgG, HRP conjugated	Horse polyclonal (Cell Signaling Technology #7076)	mouse IgG heavy and light chain	1:5000
anti-rabbit IgG, HRP conjugated	Goat polyclonal (Cell Signaling Technology #7074)	rabbit IgG heavy and light chain	1:5000
anti-goat IgG, HRP conjugated	Donkey polyclonal (Jackson ImmunoResearch #705-035-147)	goat IgG heavy and light chain	1:5000
